# Validating the APACHE IV score in predicting length of stay in the intensive care unit among patients with sepsis

**DOI:** 10.1038/s41598-023-33173-4

**Published:** 2023-04-11

**Authors:** Kinley Zangmo, Bodin Khwannimit

**Affiliations:** 1 Department of Anesthesiology, Jigme Dorji Wangchuk National Referral Hospital, 11001 Thimphu, Bhutan; 2 Division of Critical Care Medicine, Department of Internal Medicine, Faculty of Medicine, Prince of Songkla University, Hat Yai, 90110 Songkhla Thailand

**Keywords:** Medical research, Outcomes research

## Abstract

The Acute Physiology and Chronic Health Evaluation (APACHE) IV model can predict the intensive care unit (ICU) length of stay (LOS) in critically ill patients. Thus, this study aimed to validate the performance of the APACHE IV score in predicting ICU LOS among patients with sepsis. This retrospective study was conducted in the medical ICU of a tertiary university between 2017 and 2020. A total of 1,039 sepsis patients were enrolled. Patients with an ICU stay of 1 and > 3 days accounted for 20.1% and 43.9%. The overall observed and APACHE IV predicted ICU LOS were 6.3 ± 6.5 and 6.8 ± 6.5, respectively. The APACHE IV slightly over-predicted ICU LOS with standardized length of stay ratio 0.95 (95% CI 0.89–1.02). The predicted ICU LOS based on the APACHE IV score was statistically longer than the observed ICU LOS (p < 0.001) and were poorly correlated (R^2^ = 0.02, p < 0.001), especially in patients with a lower severity of illness. In conclusions the APACHE IV model poorly predicted ICU LOS in patients with sepsis. The APACHE IV score needs to be modified or we need to make a new specific model to predict ICU stays in patients with sepsis.

## Introduction

Sepsis is a major healthcare problem worldwide and is one of the most common conditions associated with admission to the intensive care unit (ICU)^1,2^. Despite advances in intensive care monitoring and managements, the mortality and cost of care of sepsis remain high^3–5^. A previous study in patients with sepsis found that the median ICU costs were $599.9 per day with a total of $2716.5 per patient. ICU costs accounted for 64.7% of the total hospital costs^4^. The costs mainly depend on the length of stay (LOS) in ICU and many factors are associated with prolonged ICU LOS, such as the cause of ICU admission, severity of illness, comorbidities and ICU management and care process^6–9^.

There is a discrepancy in LOS among ICUs that persists after adjusting for patients’ risk factors and severity of illness. Therefore, it is important if we could predict ICU LOS in critically ill patients. Comparing risk-adjusted ICU LOS between ICUs may prove supportive to risk-adjusted mortality and be used as benchmarks for assessing differences in ICU stay between facilities and examining changes in ICUs performance overtime. The Acute Physiology and Chronic Health Evaluation (APACHE) III model was the first severity-adjusted model to predict ICU LOS.

The APACHE IV model is an updated version for predicting ICU LOS in critically ill patients. This model scores demographic data, admission diagnosis, and physiological derangements and used multivariate linear regression to predict hospital mortality and ICU LOS for each patient. The APACHE IV was developed using data from 69,652 patients admitted to 104 ICUs in the USA and then validated using data from 46,517 patients^10^. Previous studies showed that the APACHE IV model provides clinically useful scores for predicting ICU LOS in critically ill patients^10,11^. However, few studies have evaluated the ability of the APACHE IV score to predict ICU LOS in patients with sepsis and a single small study found that the APACHE IV model poorly predicted ICU LOS in patients with sepsis^3^. The study aimed to validate the performance of APACHE IV for predicting ICU LOS in patients with sepsis.

## Materials and methods

This retrospective cohort study was conducted in the medical ICU of a tertiary referral and university hospital of Prince of Songkla University, Thailand. This study was approved by the Human Research Ethic Committee of Faculty of Medicine, Prince of Songkla University (REC.64-608-14-3) and was conducted under the ethical principles of the Declaration of Helsinki. The written informed consent was waived by Human Research Ethic Committee of Faculty of Medicine, Prince of Songkla University (REC.64-608-14-3) due to study design.

All patients with sepsis who were admitted to our ICU between 2017 and 2020 were enrolled to this study. The inclusion criteria were patients diagnosed with sepsis by the Sepsis-3 definition (defined by Sequential Organ Failure Assessment (SOFA) score > 2)^12^, age ≥ 18 year and ICU stay ≥ 4 h (as APACHE IV criteria)^10,13^. Septic shock was defined by sepsis requiring vasoactive agents to maintain mean arterial pressure ≥ 65 mm Hg and serum lactate ≥ 2 mmol/L^12^. Patients who were readmitted to the ICU during the study period were excluded. Patients were followed up until they were discharged from the ICU, and the observed ICU LOS were recorded. ICU LOS, defined in day, was defined as the time of admission to discharge from the ICU. The LOS truncated at 30 days to minimize the impact of outliers, as in previous study^11^. Our ICU covered by four, full-time, board-certified intensivists, who make the decision for discharge the patients from the ICU. In briefly, based on our ICU protocol, discharge from the ICU is indicated if patients’ vital signs are stable, no require invasive hemodynamic monitoring or life organ supports and available nursing care at wards.

Patient data were collected from the database of severity scores and sepsis registry of Division of Critical Care Medicine, Department of Internal Medicine. The variables used to calculate the APACHE IV score and Simplified Acute Physiology Score (SAPS) II^14^ were collected, including age, sex, comorbidities, type of ICU admission, admission source, LOS before ICU admission, site of infection, mechanical ventilator used, date of ICU admission and discharge or death, hemodynamic data (body temperature, heart rate, respiratory rate, blood pressure, urine output), blood chemistries (hematocrit, white blood cell count, platelets, serum albumin, bilirubin, glucose, sodium, potassium, bicarbonate, BUN, creatinine, arterial blood gas, lactate), and Glasgow Coma Score^10^. Hemodynamic and blood chemistry data of the APACHE IV and SAPS II model were based on the worst values within the first 24 h after ICU admission. The APACHE IV score and predicted hospital and ICU LOS was calculated using a calculator from https://intensivecarenetwork.com/Calculators/Files/Apache4.html.

Qualitative data were summarized as numbers and percentages and quantitative data were expressed as mean ± standard deviation or median [interquartile range], as appropriate. The chi-square test was used to compare categorical variables and Student’s t-test or Wilcoxon rank-sum test were used to compare continuous variables. Multiple methods were used to assess the performance of the APACHE IV model in predicting ICU LOS. The correspondence between mean observed ICU LOS and predicted ICU LOS was evaluated using a paired Student’s t-test to compare the whole population and for subgroups analysis (age, sex, type of admission, sepsis and septic shock, source of infection and severity of patients). We measured the variance in LOS explained by the model and calculated the coefficient of determination (R^2^) equal to the square of the correlation coefficient between the individual predicted ICU LOS and the observed ICU LOS^10,11^. The performance of APACHE IV to predicted ICU LOS was determined by calculating the standardized LOS ratio that defined by the mean observed ICU LOS divided by the mean predicted ICU LOS. 95%confidence intervals (CIs) were calculated by the Fieller method^11^. We determined the association between actual and predicted ICU LOS and the severity of illness assessed using the APACHE IV score. The patients were divided into deciles of predicted ICU LOS and used the paired Student’s-t test and calibration curves to compare mean predicted ICU LOS to actual ICU LOS^11^. The calibration graph was displaying by mean observed and mean predicted ICU LOS throughout the range of observed values^10,11^. We also compared the mean observed and predicted ICU LOS across deciles of the predicted LOS using APACHE IV. The accuracy of the APACHE IV model for predicting ICU stays more than 1, 3 and 7 days were assessed using the area under the receiving operating characteristic curve (AUC). We also compared the AUC of APACHE IV with SAPS II and SOFA score for predicting ICU stays by using the method of Delong et al.^15^. Statistically significant was set at p < 0.05 and statistical analyses were performed using Stata 15 software.

## Results

A total of 1039 patients with sepsis were enrolled in this study. Septic shock was identified in 786 patients (75.6%). The demographic and clinical characteristic of patients are shown in Table 1. Most patients (89%) required mechanical ventilator support.

**Table 1:** Demographic and clinical characteristics of patients with sepsis.

Characteristics	
Age, years	61.9 ± 19.1
Male, n (%)	594 (57.2)
Source of admission, n (%)	
Wards	431 (41.4)
ED	434 (41.8)
Refer	174 (16.8)
Septic shock	786 (75.6)
Community-acquired infection, n (%)	668 (64.3)
Site of infection	
Respiratory tract	607 (58.5)
Urinary tract	106 (10.2)
Gastrointestinal	107 (10.3)
Primary bacteremia	104 (10.0)
Others	113 (10.9)
SAPS II	54.1 ± 20.5
SOFA score	2.5 ± 1.7
APACHE IV score	87.3 ± 39.8
Observed ICU LOS (days)	6.4 ± 6.5
Hospital LOS (days)	27.0 ± 31.1
Observed hospital mortality, n (%)	437 (42.1)
APACHE IV predicted ICU LOS (days)	6.8 ± 1.3
APACHE IV predicted hospital mortality (%)	46.2 ± 29.1

*APACHE* acute physiology and chronic health evaluation, *ED* emergency department, *LOS* length of stay, *ICU* intensive care unit, *SAPS* simplified acute physiology score, *SOFA* sequential organ failure assessment.

**Figure 1:**
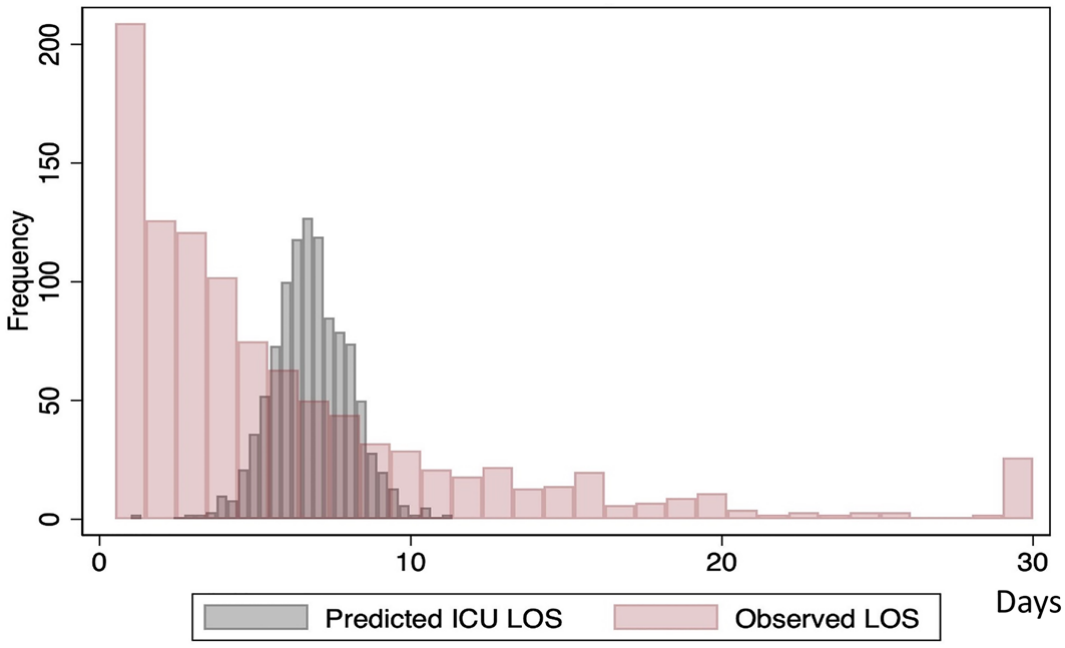
Distribution of observed and APACHE IV predicted ICU length of stay. *APACHE* acute physiology and chronic health evaluation, *ICU* intensive care unit, *LOS* length of stay.

**Figure 2:**
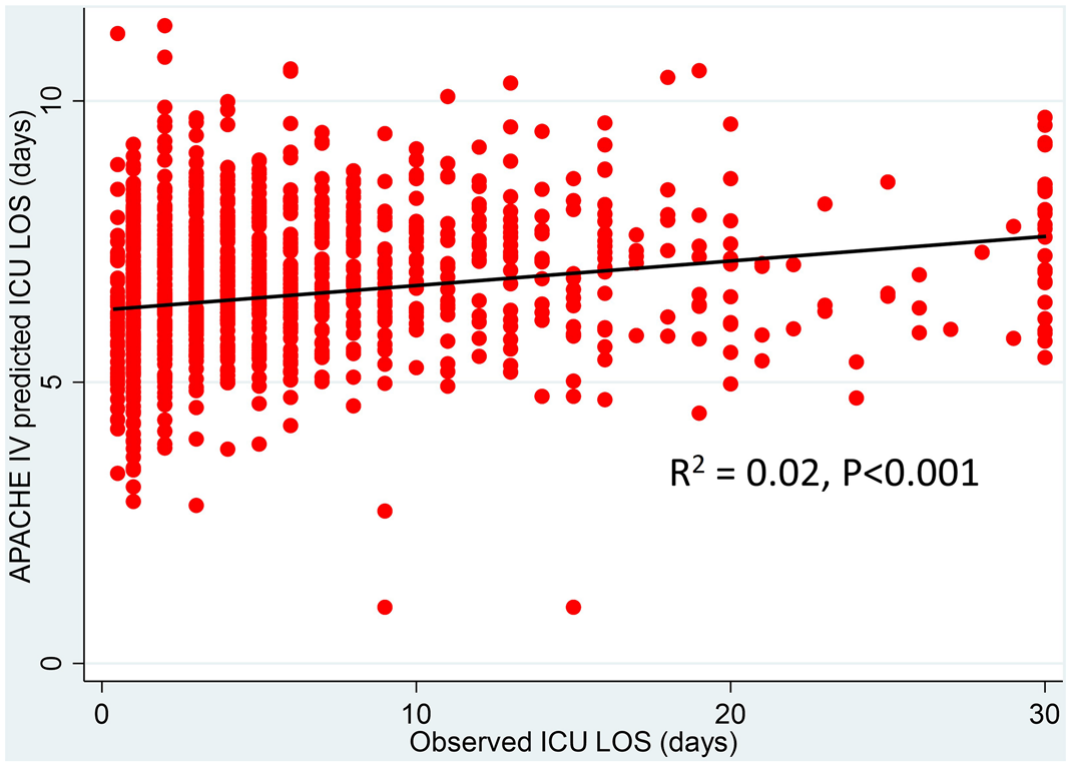
Correlation between observed ICU length of stay and APACHE IV predicted length of stay. *APACHE* acute physiology and chronic health evaluation, *ICU* intensive care unit, *LOS* length of stay.

**Figure 3:**
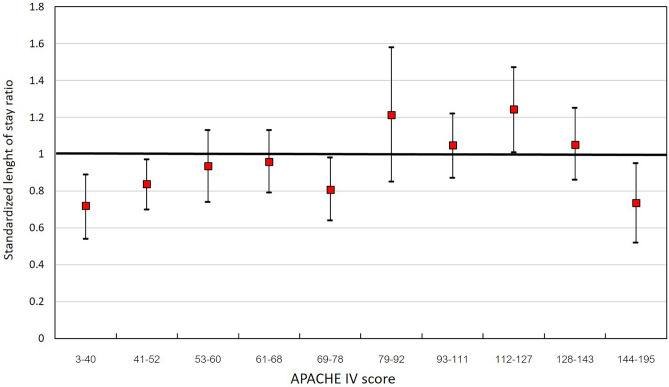
Standardized ICU length of stay ratio with 95%CI stratified by the APACHE IV score. *APACHE* acute physiology and chronic health evaluation, *CI* confidence interval, *ICU* intensive care unit.

The distribution of observed and APACHE IV predicted ICU LOS was showed in Fig. 1. Patients with an ICU stay of 1, > 3 and > 7 days accounted for 20.1%, 43.9% and 28.2% of patients with sepsis, respectively. The APACHE IV predicted mean and median ICU LOS was 6.8 ± 1.3 and 6.7 [6–7.6] days, while the overall mean and median ICU LOS were 6.4 ± 6.5 and 4 [2–8] days, respectively. The predicted ICU LOS base on the APACHE IV score were significantly longer than the observed ICU LOS (p < 0.001). Linear regression analysis found that the APACHE IV predicted ICU LOS poorly correlated with observed ICU LOS (R^2^ = 0.02, p < 0.001, Fig. 2) and the APACHE IV score slightly over-predicted ICU LOS, with a standardized LOS ratio of 0.95 (95% CI 0.89–1.02). When sepsis patients were categorized according to the APACHE IV score, it significantly exceeded the predicted ICU LOS in sepsis patients with an APACHE IV < 52 (Fig. 3). Overall, the APACHE IV score trended to over-predicted ICU LOS, especially in patients with a lower severity of illness. When the APACHE IV score ranges from to 3–40 and 41–52, the APACHE IV predicted ICU LOS with a standardized LOS of 0.72 (95% CI 0.54–0.89) and 0.84 (95% CI 0.7–0.97), respectively.

**Figure 4:**
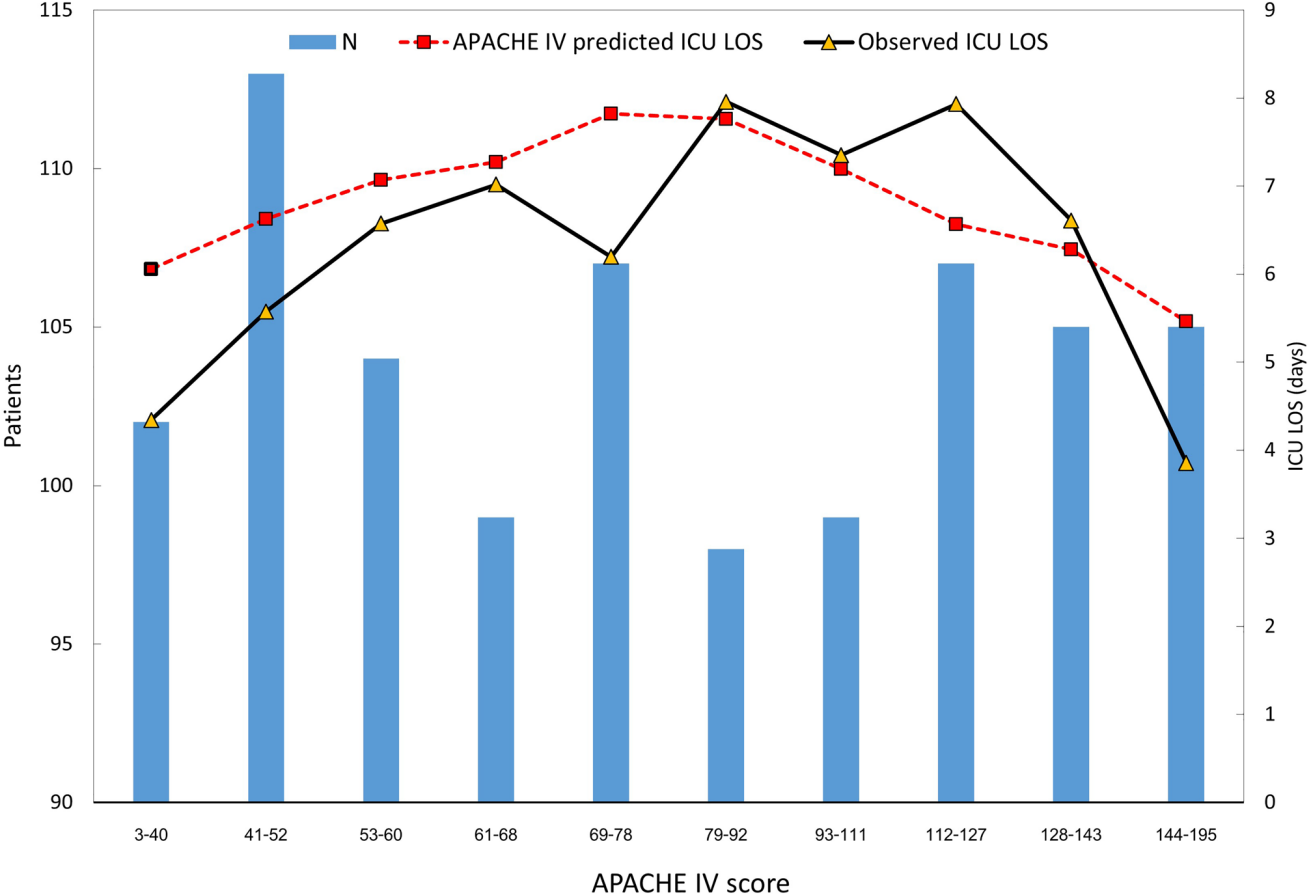
Associated between ICU length of stay and distribution of APACHE IV score. *APACHE* acute physiology and chronic health evaluation, *ICU* intensive care unit, *LOS* length of stay.

ICU LOS was correlated with patient severity. Figure 4 shows the association between APACHE IV score and observed and predicted ICU LOS. Increasing disease severity using the APACHE IV score is associated with an increase in both the actual and predicted ICU LOS. However, when the APACHE IV score exceeded 78, actual ICU LOS gradually declined.

**Figure 5:**
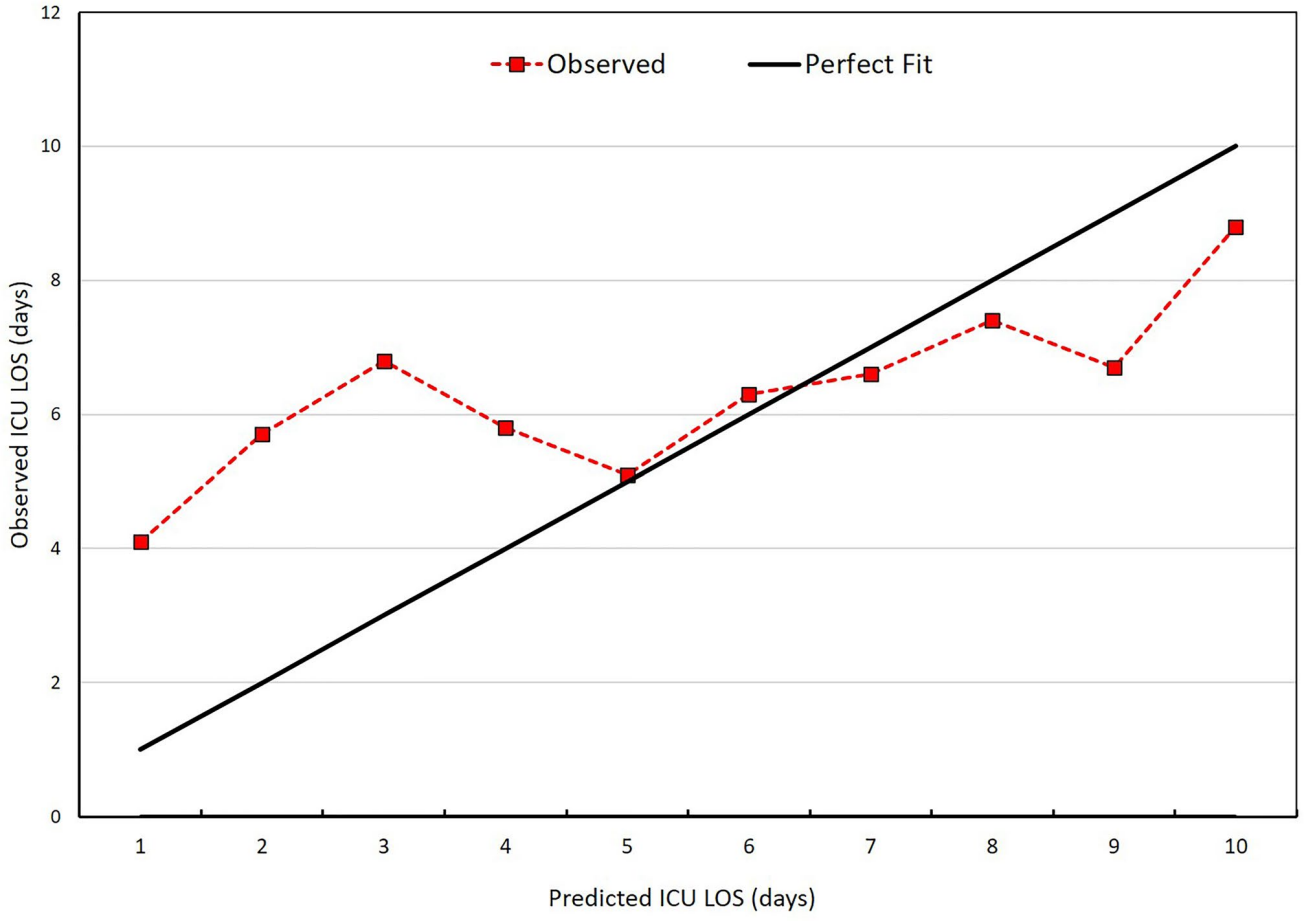
Calibration curve comparing mean observed and mean APACHE IV predicted ICU length of stay for 10 equal-sized groups. *APACHE* acute physiology and chronic health evaluation, *ICU* intensive care unit, *LOS* length of stay.

The impact of the predicted ICU LOS based on the decile of the predicted ICU LOS is shown in Table 2. For each decile of predicted ICU LOS, the difference between the mean observed and APACHE IV predicted ICU LOS differed significantly between the two deciles. The 5^th^ and 9^th^ decile sepsis patients had a significant difference between observed and predicted ICU LOS of 1.5 and 1.42 days, respectively. The calibration curve is presented in Fig. 5 and demonstrates that APACHE IV had a poor fit for predicting ICU LOS across multiple deciles.

**Table 2:** Difference between observed and predicted ICU LOS across decile of predicted LOS by APACHE IV.

Decile of predicted ICU LOS*%	Patients, No	Mean observed ICU LOS, days	Mean predicted ICU LOS, days	Mean difference observed-predicted ICU LOS, days	Ratio observed-predicted ICU LOS, days	P-value**
0–10	103	4.1	4.6	0.48	1.05	0.32
11–20	104	5.7	5.6	− 0.18	1.03	0.77
21–30	104	6.8	6	− 0.78	1.1	0.29
31–40	104	5.8	6.3	0.47	0.92	0.4
41–50	104	5.1	6.6	1.5	0.77	0.001
51–60	104	6.3	6.9	0.57	0.92	0.37
61–70	104	6.6	7.2	0.61	0.92	0.33
71–80	104	7.4	7.6	0.22	0.97	0.72
81–90	104	6.7	8.1	1.42	0.82	0.02
91–100	104	8.8	9.1	0.23	0.98	0.78

*APACHE* acute physiology and chronic health evaluation, *LOS* length of stay, *ICU* intensive care unit.

*Population sorted by increasing predicted risk and the split into deciles.

**Based on paired Student t-test.

**Figure 6:**
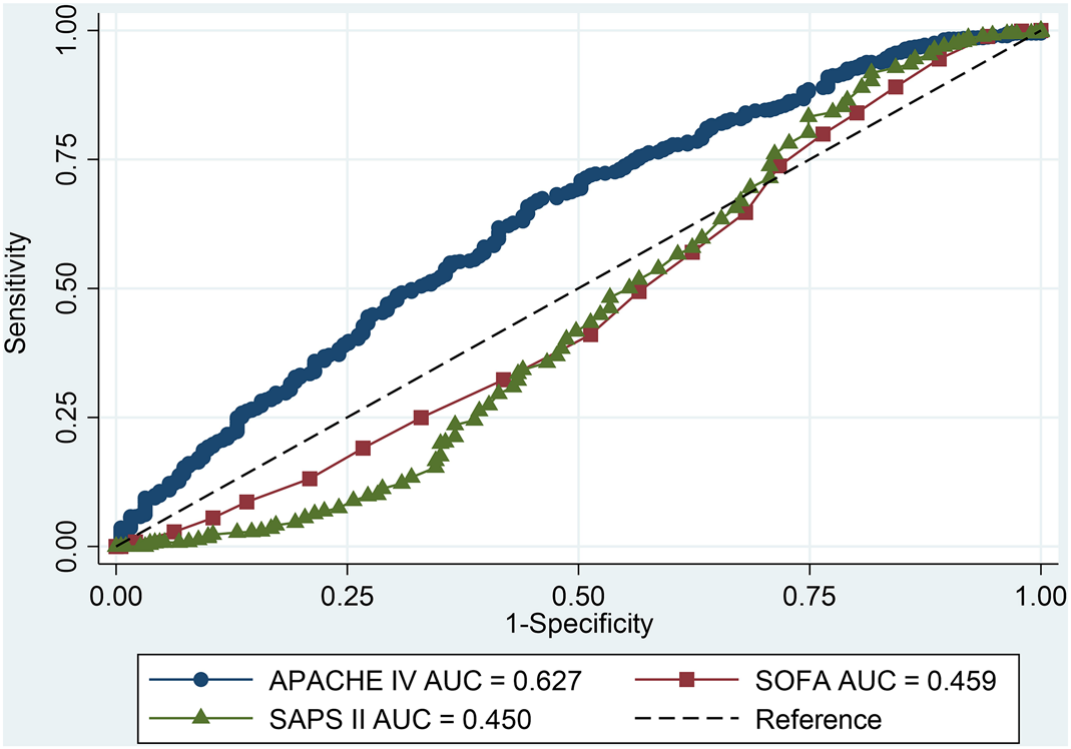
Comparison predictive ability of APACHE IV, SAPS II and SOFA score for predicting ICU LOS > 1 day. *APACHE* acute physiology and chronic health evaluation, *AUC* area under the receiving operating characteristic curve, *ICU* intensive care unit, *LOS* length of stay, *SAPS* simplified acute physiology score, *SOFA* sequential organ failure assessment.

There were 830, 583, and 293 sepsis patients with ICU stays of > 1, 3, and 7 days, respectively. The accuracy of the APACHE IV model in predicting ICU LOS > 1, 3, and 7 days was 0.627 (95% CI 0.583–0.670), 0.587 (95% CI 0.552–0.622) and 0.582 (95% CI 0.544–0.620), respectively. The APACHE IV ≥ 50 had a sensitivity 82.9% and specificity 20.1% for predicting ICU LOS > 1 day. The APACHE IV had a higher AUC for predicting ICU LOS > 1 day than SAPS II (AUC 0.450, 95% CI 0.397–0.503, p < 0.001) and SOFA score (AUC 0.459, 95% CI 0.412–0.506, p < 0.001) (Fig. 6). Moreover, the AUC for predicting ICU LOS > 3 days of APACHE IV was significantly higher than SAPS II (AUC 0.525, 95% CI 0.487–0.563, p = 0.03) and SOFA score (AUC 0.530, 95% CI 0.494–0.566, p = 0.03).

Subgroup comparison for observed and predicted ICU LOS showed that APACHE IV predicted LOS differed significantly among female patients, patients from the emergency department, referred patients, patients without septic shock, patients with community-acquired infection, patients who survived in the ICU, and younger age groups (15–50 years) (Table 3).

**Table 3:** Differences between mean observed ICU LOS and APACHE IV predicted ICU LOS in the subgroup analysis.

Categories	N	Observed ICU LOS (mean)	APACHE IV predicted ICU LOS (mean)	Mean difference	Mean ratio of ICU LOS	P-value
Total	1,039	6.4	6.8	0.45	0.95	0.02
Female	445	6.0	6.8	0.76	0.89	0.006
Age						
15–50	257	6.45	7.18	0.73	0.90	0.06
51–64	261	6.48	6.93	0.45	0.95	0.28
65–76	255	6.23	6.79	0.56	0.91	0.15
> 76	266	6.33	6.33	0.003	1.06	0.99
Source of admission						
Wards	431	7.36	7.08	− 0.28	1.04	0.39
ED	434	5.76	6.39	0.63	0.96	0.03
Refer	174	5.45	7.15	1.70	0.74	< 0.001
Type of sepsis						
Septic shock	786	6.73	6.83	0.09	1.01	0.69
Non-septic shock	253	5.25	6.70	1.48	0.78	< 0.001
Type of infection						
Community	668	5.76	6.59	0.83	0.89	< 0.001
Nosocomial	371	7.47	7.19	− 0.28	1.07	0.45
Outcome						
ICU death	285	6.36	6.25	− 0.10	1.02	0.78
ICU survive	754	6.38	7.01	0.63	0.93	0.006
APACHE IV score						
3–55	319	5.54	6.59	1.05	0.83	0.001
56–77	206	6.61	7.56	0.95	0.88	0.04
78–120	304	7.79	7.16	− 0.63	1.17	0.10
> 120	210	5.35	5.87	0.52	0.90	0.22

*APACHE* acute physiology and chronic health evaluation, *ED* emergency department, *LOS* length of stay, *ICU* intensive care unit.

## Discussion

Our single-center retrospective study on the validation of the APACHE IV model to predict ICU LOS in sepsis patients in a medical ICU showed that predicted ICU LOS by APACHE IV poorly correlated with observed ICU LOS. The model trended to over-predict ICU LOS in patients with sepsis and some subgroup of sepsis patients.

Progression of the primary disease, underlying comorbidities, treatment complications, and hospital-acquired infections tend to prolong ICU LOS in patients with sepsis. Prolonged stay in the ICU affects not only patients but also increases the use of hospital and ICU resources, which contributes to a higher cost of care. ICUs are one of the most expensive healthcare services, as large spaces, experienced and skilled healthcare personnel, advanced monitoring equipment and organ support is required^4^.

Many studies have shown that increased ICU LOS is associated with a higher cost of care^16–19^. Furthermore, ICU costs, including median cost, daily cost, and medications, were higher in patients with septic shock than in patients with sepsis^4^. Previous studies demonstrated that the ICU cost was higher in non-survivors and patients with a longer ICU LOS^4,16^. Prolonged ICU stay was also associated with an increased risk for nosocomial-acquired infection or delirium, with a higher risk of hospital death and higher cost of health care services^7^.

Many prognostic scoring systems have been developed to allow for discrimination between survivors and non-survivors in the ICU and several severity scoring models have been proven to accurately predict outcomes in critically ill patients^10,11,13,20–23^. The APACHE model is one of the most commonly use severity scoring system in ICU and defines the severity of illness according to the degree of physiological derangement as well as the chronic health status of the patients^13^. The APACHE IV score is the latest version of the APACHE models that not only predicts mortality but also ICU LOS^10^. A previous study in our ICU showed that the APACHE IV score had better discrimination in predicting mortality than other severity scores in patients with sepsis^20^. However, few studies have determined the ability of the APACHE IV model to predict ICU LOS^3,23^. Zimmerman et al. found that the APACHE IV model significantly under-predicted ICU stay but mentioned that APACHE IV model is clinically useful in ICU patients groups^10^. A multicenter retrospective study demonstrated that the APACHE IV score is a superior tool for predicting ICU LOS when compared with the Mortality Prediction Model (MPM)-0 and SAPS II^11^. A single pilot study predicting LOS using the APACHE IV score in patients with severe sepsis found that the APACHE IV score underpredicted ICU LOS, especially in patients receiving blood transfusion, any procedure and frequent dialysis^3^. Our study found that APACHE IV tended to overpredict ICU LOS in patients with sepsis. However, both studies confirmed that APACHE IV was poorly calibrated to predict ICU LOS in patients with sepsis.

The APACHE IV ICU LOS benchmarks are clinically useful for the assessment and comparison for patient groups across ICUs, but not for predicting ICU LOS for individual patients. Several factors affected individual patients’ ICU stays including patients, structural, and managerial factors. Patient factors, such as underlying diseases, response to medications treatment, and development of complications such as ventilator-associated pneumonia, catheter-related bloodstream infection, and critical illness polyneuropathy, were found to prolong ICU stays. Structural factors that might affect ICU stay include variations in the type of ICU physician staffing or intensivists, nurse-to-patients’ ratio, open or close ICU unit, and availability of intermediate care or step-down unit or ward beds. Managerial factors include differences in ICU policies, use of standard protocols care such as analgesic and sedation and weaning protocols^19,24–26^.

The suboptimal performance of APACHE IV for predicting ICU LOS may be from the difference in case-mix and ICU care process. In addition, model performance deteriorates over time from several causes such as the improvement of health care, extension of life expectancy and the new emerging disease. Therefore, external validation should be performed before used the APACHE IV score for ICU benchmark. The poor ability of the APACHE IV to predict ICU LOS might be improved by customized the model. Customization was performed by re-estimating the coefficients of the original variables or by adding new variables such as hospital discharge policies and patient complications during ICU stay. Furthermore, machine learning techniques can be used to improve the performance of the severity scoring system. Previous studies found that machine learning algorithms improved ability to predict hospital mortality of ICU patients when compared with conventional severity model^27^ and also a good performance to predict short and long length of ICU stay^28^.

Some of the limitations of our study were its retrospective design which could have led to selection bias due to the potential for miscoding and missing data. Second, it was conducted in a single hospital and confined to the medical ICU for specific patients with sepsis, therefore, the findings of our study may not be applicable to other ICU populations and may not represent patients with sepsis in other hospitals. Third, the APACHE IV scoring model was developed using a cohort of mixed critically ill patients; it was not specifically designed for patients with sepsis. Therefore, this scoring model may not be appropriate for predicting ICU LOS in critically ill patients with sepsis. Fourth, the length of stay in the ICU is influenced by various factors, such as hospital and ICU protocols, ICU infrastructure, and end-of-patient care, which are different in different hospitals and institutions.

## Conclusion

It is important to determine both patient prognosis and LOS of critically ill patients in the ICU. Our study demonstrated that the APACHE IV model poorly predicted ICU LOS in patients with sepsis. The APACHE IV score needs to be updated or modified or to include a new specific severity-adjusted LOS to predict ICU stays in patients with sepsis.

## Data Availability

The datasets used and/or analyzed during the current study available from the corresponding author on reasonable request.
